# Novel Cytokinin Derivatives Do Not Show Negative Effects on Root Growth and Proliferation in Submicromolar Range

**DOI:** 10.1371/journal.pone.0039293

**Published:** 2012-06-18

**Authors:** Kateřina Podlešáková, David Zalabák, Mária Čudejková, Ondřej Plíhal, Lucie Szüčová, Karel Doležal, Lukáš Spíchal, Miroslav Strnad, Petr Galuszka

**Affiliations:** 1 Department of Biochemistry, Faculty of Science, Palacký University, Olomouc, Czech Republic; 2 Centre of the Region Haná for Biotechnological and Agricultural Research, Faculty of Science, Palacký University, Olomouc, Czech Republic; Iwate University, Japan

## Abstract

**Background:**

When applied to a nutrition solution or agar media, the non-substituted aromatic cytokinins caused thickening and shortening of the primary root, had an inhibitory effect on lateral root branching, and even showed some negative effects on development of the aerial part at as low as a 10 nanomolar concentration. Novel analogues of aromatic cytokinins ranking among topolins substituted on *N9*-atom of adenine by tetrahydropyranyl or 4-chlorobutyl group have been prepared and tested in standardized cytokinin bioassays [1]. Those showing comparable activities with *N^6^*-benzylaminopurine were further tested *in planta*.

**Methodology/Principal Findings:**

The main aim of the study was to explain molecular mechanism of function of novel cytokinin derivatives on plant development. Precise quantification of cytokinin content and profiling of genes involved in cytokinin metabolism and perception in treated plants revealed several aspects of different action of *m-*methoxytopolin base and its substituted derivative on plant development. In contrast to standard cytokinins, *N9*- tetrahydropyranyl derivative of *m-*topolin and its methoxy-counterpart showed the negative effects on root development only at three orders of magnitude higher concentrations. Moreover, the methoxy-derivative demonstrates a positive effect on lateral root branching and leaf emerging in a nanomolar range of concentrations, in comparison with untreated plants.

**Conclusions/Significance:**

Tetrahydropyranyl substitution at *N9*-position of cytokinin purine ring significantly enhances acropetal transport of a given cytokinins. Together with the methoxy-substitution, impedes accumulation of non-active cytokinin glucoside forms in roots, allows gradual release of the active base, and has a significant effect on the distribution and amount of endogenous isoprenoid cytokinins in different plant tissues. The utilization of novel aromatic cytokinin derivatives can distinctively improve expected hormonal effects in plant propagation techniques in the future.

## Introduction

Besides auxins, cytokinins (CKs) are the most substantial group of plant hormones with the ability to regulate plant growth, organ development and coordinate many physiological processes. While auxins found practical utilization as a rooting agent for various cultivation techniques, CKs have so far acquired the biggest potential in agricultural technologies as components of different culture media for plant regeneration *in vitro*. Biotechnology of tissue cultures is exploited especially for rapid and cost-effective propagation of ornamental plants, some crops such as banana and many endangered species. In micropropagation, CKs are added to the media to keep proportional organ differentiation in coordination with auxins. By the accurate regulation of CK levels through medium, desired traits of propagated plants can be achieved such as dwarf and enormous shoot branching phenotypes [e.g. 2,3], increased resistance to phytopathogens [4] or increased level of somaclonal variation [5].

Presently, *N^6^*-benzylaminopurine (BAP) and kinetin are the most widely used CKs in micropropagation techniques. Firstly, it is due to their high biological activity, accessibility and low-cost. In contrast to isoprenoid CKs, they have higher *in vivo* stability due to a significantly higher resistance to oxidative degradation [6], which allows dropping the effective concentration down to nanomolar levels. BAP can occur naturally in the form of benzyl side-chain substituted derivatives (topolins). It was shown several times that application of BAP with hydroxyl or methoxy-group in *meta* position can improve properties of explants in many parameters contrary to non-substitued BAP [7]–[9]. The *meta* hydroxy- substituted BAP (*m-*topolin) shows a generally higher CK activity in standardized CK bioassays than the *ortho* and *para* isomers and comparable activity to BAP [10], [11]. The potent effect of *m-*topolin was confirmed in a CK receptor test [11], [12] and can be attributed, besides the molecule conformation, also to the considerable low affinity to CK degradation enzymes [1].

Apart from the *N^6^*-substituent, CK molecules can be modified on purine ring usually by glycosylation resulting prevailingly in reduced biological activity. Hence, free bases of aromatic CKs are most frequently applied to culture media. However, their application is often connected with some negative effects. The application of BAP to some extent can have an inhibitory effect on root formation and heterogeneity in growth [13]. Some authors explain the root growth inhibition by extensive accumulation of non-active CK 9-glucosides [13] or activation of ethylene production [14]. Applied CK, in concentration higher than 100 nM, is able to accelerate ethylene biosynthesis in Arabidopsis and pea seedlings [15], [16]. However, ethylene production or perception inhibitors did not rescue root elongation completely after exogenous BAP application [15]. Therefore, it is likely that CKs can inhibit root elongation by another mechanism which is not mediated via ethylene. Recently, it was shown that BAP itself, independently on ethylene production, dramatically reduces mitotic activity in proximal meristem of roots, but BAP activated overproduction of ethylene reduces cell elongation and overall root growth probably by disturbance of auxin action [17], [18].

To prevent the negative effect of glycosylation, treatments with CK derivatives substituted at *N9*-position were tested and the metabolism of these compounds was followed. Although in most cases the substitutions led to a significant loss of CK activity [19], [20] or to rapid degradation of CK derivatives [19], [21], two modifications, *N9*-tetrahydropyranyl (THP) and *N9*-tetrahydrofuranyl, kept comparable activity to the parental compound in different biotests [19], [22], [23]. Firstly, the positive effect of treatment with CK tetrahydropyranyl derivative was shown in several varieties of grapevine [24]. Dipping of flower cluster in solution of 6-(benzylamino)-9-(2-tetrahydropyranyl)purine (BA9THPP) dramatically promotes fruit setting. Nevertheless, the authors did not compare results acquired with BA9THPP to the effect of free BAP treatment, and as such an application has not found any practical use. The later works clearly showed that either *N9*-methyl, *N9*-(4-chlorobutyl) or *N9*-THP substituents can be dealkylated and do not protect CK molecule against glycosylation *in vivo* [7], [19], [21], [25]. Furthermore, it was not confirmed that the uptake of BA9THPP into plant tissue is enhanced compared to free BAP [19] and THP derivatives have a higher stability against enzymatic cleavage of *N^6^*-side-chain [1]. Prompt *N9*-glucosylation following the CK uptake from media largely compromises the CK translocation to other developing organs; the only well transportable form of BAP was found to be its *N9*-ribosides [13].

Surprisingly, it was shown that a 20****micromolar application of *m-*topolin does not have a significant negative effect on root growth contrary to equimolar application of BAP or its *N9*-THP and *N9*-glucoside form [7]. The authors explained this phenomenon by proposing that free hydroxy group on the benzyl ring might be subjected to extensive glucosylation. Hence, we synthesized in our previous work [1] an array of synthetic *N9*-derivatives based on hydroxy- and methoxy-BAP, which combine both types of substitutions with a positive effect on *in vitro* explants as mentioned above. Here we present their impact on phenotype of two studied model plants after exogenous treatments, their perception via CK receptors, a detailed metabolic study *in planta*, distribution of these compounds through the plant body and influence on endogenous CK metabolism.

## Results

### Influence of 3-methoxy(-6-benzylamino)purine on plant morphology

**Figure 1:**
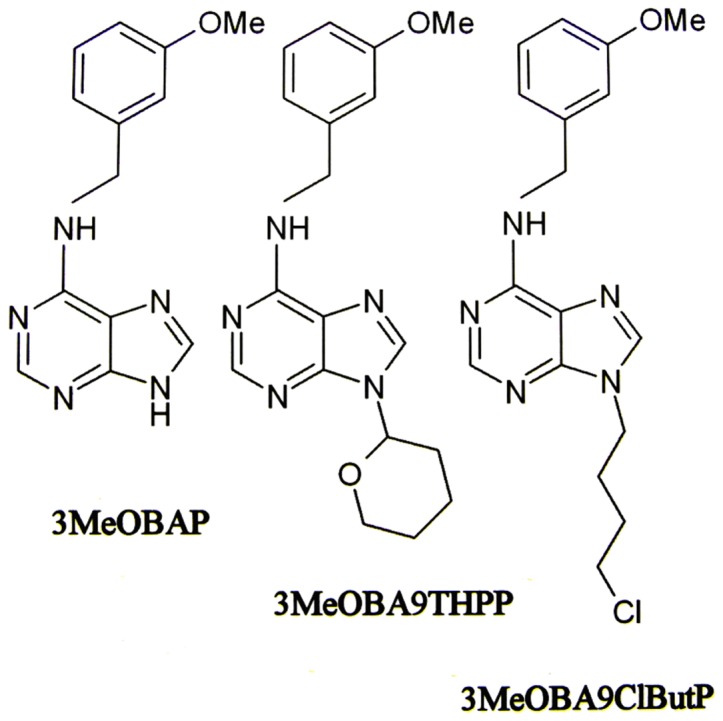
Structure of 3MeOBAP and its novel *N9*-derivatives used in the study.

**Figure 2:**
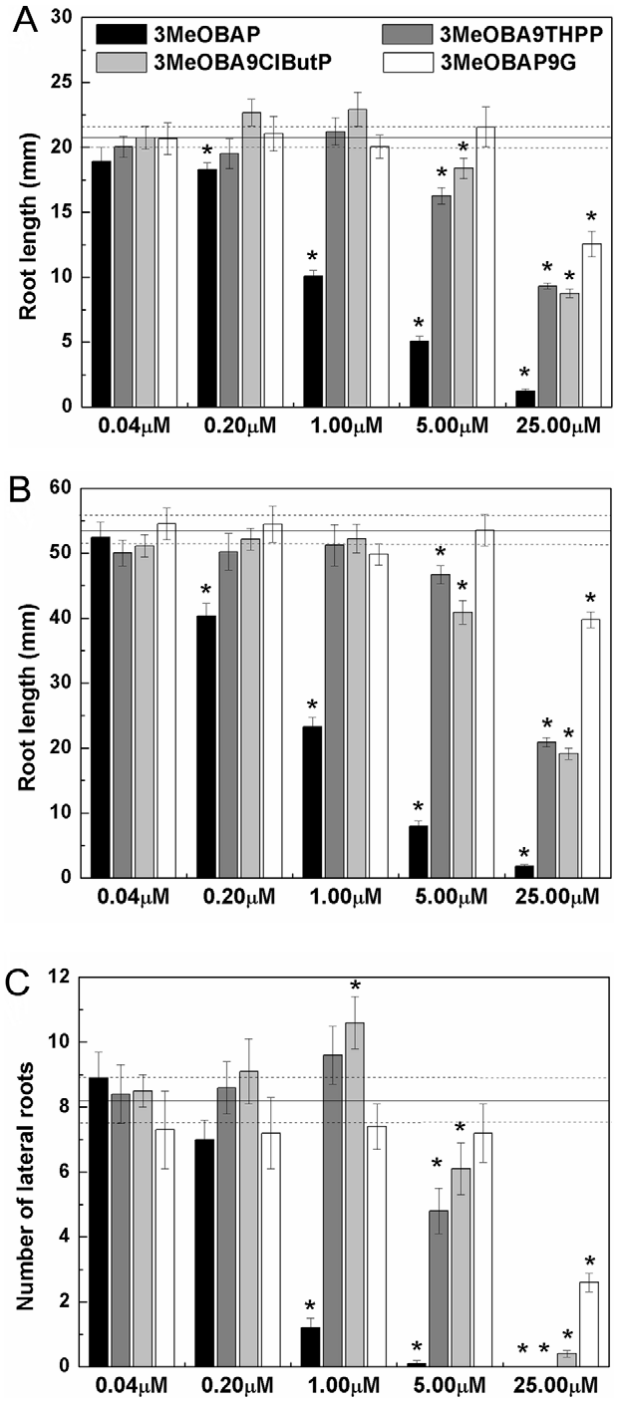
Primary root length and number of lateral roots of Arabidopsis seedlings grown on media supplemented with 3MeOBAP derivatives. Root length was determined in 7-day-old (**A**) and 14-day-old seedlings (**B**), and the number of emerged lateral roots in 14-day-old seedlings (**C**). Each value is an average ±SD of measurements in at least 90 plantlets grown on 9 independent vertical dishes. Solid line indicates measured parameters and dashed lines indicate SD of control plants. Asterisks indicate the significant difference (*P* ≤ 0.05) between untreated and treated plants according to Student's unpaired t-test.

**Figure 3:**
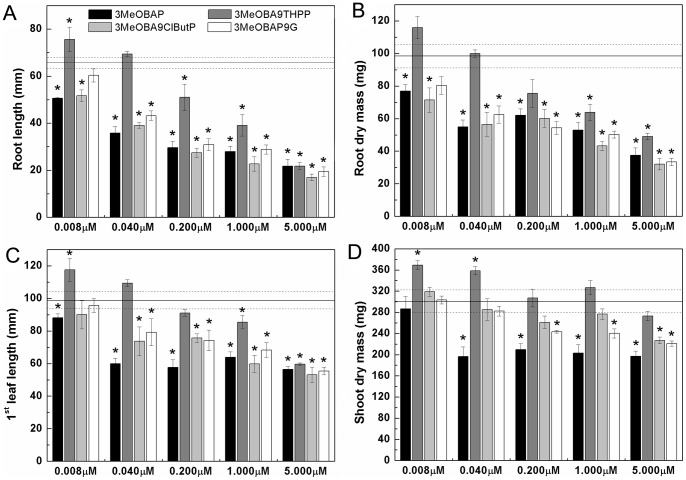
The effects of 3MeOBAP *N9*-modifications on length of the primary root (A) and the first emerged leaf (C) in 7-day-old maize seedlings and biomass production of the root system (B) and the aerial part (D). 4-day-old maize plantlets were cultivated for 5 days in nutrient solution supplemented with CKs. Each value is an average ±SD of measurements of at least 120 seedlings grown in 12 independent hydroponic boxes. Solid line indicates measured parameters and dashed lines indicate SD of control plants. Asterisks indicate the significant difference (*P* ≤ 0.05) between untreated and treated plants according to Student's unpaired t-test.

The application of 3-methoxy(-6-benzylamino-9-tetrahydropyran-2-yl)purine (3MeOBA9THPP), 3-methoxy(6-benzylamino-9-chlorobutyl)purine (3MeOBA9ClButP; Figure 1) and 3-hydroxy(-6-benzylamino-9-glucosyl)purine (3MeOBAP9G) into the MS media did not have any distinct inhibitory effect on elongation of the Arabidopsis primary root and lateral root branching in a broad range of concentrations from 0.04 to 5 μM compared to significant negative/inhibitory effect of 3-methoxy(-6-benzylamino)purine (3MeOBAP) application in the same concentration range (Figure S1, 3AB, 2AB). On the contrary, CKs with both types of *N9*-protection have slightly increased the number of emerged lateral roots (Figure 2C). 3MeOBAP9G has not had any significant effect on Arabidopsis morphology up to 5 μM concentration.

Similarly to Arabidopsis plantlets, the addition of 3MeOBA9THPP up to 1 μM concentration lacked the inhibitory effect on root elongation and lateral root growth in maize (Figure S2). However, treatments with 3MeOBA9ClButP and 3MeOBAP9G had an inhibitory effect comparable with the effect of free base. A positive influence of 3MeOBA9THPP on lateral root growth and elongation of primary root was observed in the nanomolar range of concentrations (8 to 40 nM, Figure 3). The positive impact of the 3MeOBA9THPP application in the nanomolar range of concentrations on overall organ growth was observed in both the roots as well as in the aerial part of seedlings; it was expressed both as root/leaf length as well as a production of the dry root/leaf mass (Figure 3).

### Activation of Arabidopsis and maize cytokinin receptors

**Figure 4:**
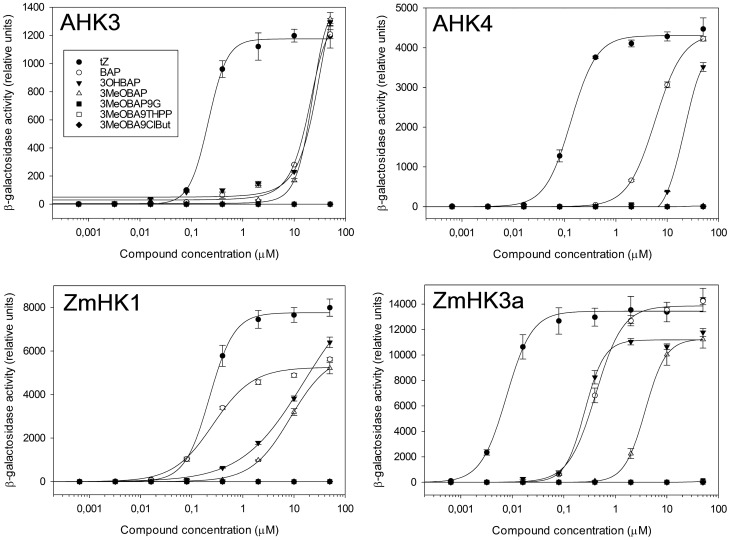
CK receptor studies. *β*-galactosidase induction in *E. coli* strain Δ*rcsC, cps::lacZ*) upon incubation with CK derivatives. Strains harboring constructs of Arabidopsis receptor AHK3 and AHK4, or maize receptor ZmHK1 and ZmHK3a were activated by the selected compounds in a dose-dependent manner. The values shown are means with ±SD (n = 3).

*N9*-derivatives showed similar responses in standardized CK bioassays as 3OHBAP and BAP. To see whether these CK derivatives are able to trigger a response in CK receptors (histidine kinases; HKs), we studied their interaction with Arabidopsis and maize receptors in the bacterial assay [26]. Particular CK receptors were overexpressed in *E.coli* strain with knock-out hybrid sensor histidine like kinase (RcsB) – structural analog of plant HK, which is involved in regulation of an extracellular polysaccharide synthesis by activating the capsular polysaccharide synthesis (cps) operon. *LacZ* gene under the control of cps promoter has been introduced into its genome. Level of *LacZ* activation expressed as conversion of 4-methylumbelliferyl β-D-galactopyranoside was estimated after addition of different CKs into bacterial growth media. The data from those experiments clearly showed that only CK free bases could induce response pathway; none of the studied *N9*-derivatives was able to activate the receptors, even at 50 µM concentration (Figure 4). Aromatic CKs were generally better ligands for ZmHKs than for AHKs. 3OHBAP triggered CK response with similar intensity as BAP in the case of two out of four studied HKs. In contrast, 3MeOBAP was neither able to induce any response in the studied receptors nor some minor activation was observed only in the high concentration range. Activation of ZmHK2 and AHK2 receptors in the bacterial assay was overall much weaker than activation of other HKs, detectable only with a high concentration of *trans-*zeatin (tZ), which is in agreement with the previous study [27].

### Metabolism and content of cytokinin in planta after exogenous cytokinin treatment

Tritium labeled stock of 3MeOBA9THPP was used for an *in vivo* metabolic study in *Zea mays*. The tissue extracts were purified and fractionated on a C18 separation column. The retention times of fractions containing detectable radioactivity were compared with the CK standards, see Table S1. The tetrahydropyranyl group at the *N9*-position of the parent CK molecule can be, to some extent, cleaved in both root and leaf tissues of maize plantlets. Released free base 3MeOBAP can be further glycosylated to both *N9*-riboside and *N9*-glucoside. Moreover, high amounts of radioactivity were reproducibly detected in an unknown derivative. The identities of all derivatives were confirmed by 1∶1 [^15^N_4_] 3MeOBA9THPP and 3MeOBA9THPP feeding. The unknown derivative proved to have a mass [M+H]^+^ = 356 and [M+H]^+^+4 = 360, which could correspond to 3MeOBA9THPP molecule with an additional hydroxyl group (Figure S3). Interestingly, 3-methoxytopolin undergoes demethylation resulting in detection of 3OHBAP and its *O-*glucoside, which accumulates predominantly in leaves.

Further CK quantification revealed that 3MeOBA9ClButP can be effectively dealkylated *in vivo* in both plant models. Unlike Arabidopsis, maize metabolism is able to detach the glucosyl moiety from 3MeOBAP9G to a similar extent as observed in the case of the THP derivative (Table 1). To confirm the *N9*-deglucosylation in maize tissues, seedlings were treated with tritium labeled BAP9G and DHZ9G and radioactivity detected in CK HPLC fractions (Figure S4). Both glucosides were susceptible to *N9*-deglucosylation to some extent; DHZ9G also underwent degradation to adenine.

**Table 1:** CK content in maize roots and Arabidopsis seedlings 72 h after exogenous application of 1 μM 3MeOBAP, 3MeOBA9THPP, 3MeOBA9ClButP and 3MeOBAP9G.

Applied cytokinin	3MeOBAP	3MeOBAPR	3MeOBA9G	3MeOBA9THPP	unknown	3MeOBA9ClButP	3OHBAP	3OHBAPR	3OHBAPR5MP	3OHBAP9G	(OG)3OHBAP
*Zea mays*											
**Control**	u.d.l.	u.d.l.	u.d.l.	u.d.l.	u.d.l.	u.d.l.	u.d.l.	u.d.l.	u.d.l.	u.d.l.	u.d.l.
**3MeOBAP**	**59.20±4.78**	3.35±0.15	238.55±35.20	u.d.l.	u.d.l.	u.d.l.	1.69±0.23	u.d.l.	u.d.l.	236.08±32.10	u.d.l.
**3MeOBA9THPP**	22.31±1.48	7.46±0.62	46.99±3.21	**61.78±2.19**	4.13±0.19	u.d.l.	2.48±1.32	u.d.l.	u.d.l.	8.67±4.10	14.81±3.20
**3MeOBA9ClButP**	31.04±2.22	21.78±3.48	338.74±29.88	u.d.l.	u.d.l.	373.13±23.04	9.76±1.54	u.d.l.	u.d.l.	117.38±24.40	10.54±2.10
**3MeOBAP9G**	8.30±0.28	2.26±0.18	**100.14±1.92**	u.d.l.	u.d.l.	u.d.l.	3.94±1.50	u.d.l.	u.d.l.	5.46±2.66	7.58±1.80
*Arabidopsis thaliana*											
**Control**	u.d.l.	u.d.l.	u.d.l.	u.d.l.	u.d.l.	u.d.l.	u.d.l.	u.d.l.	u.d.l.	u.d.l.	u.d.l.
**3MeOBAP**	**78.54±4.23**	14.34±1.56	11.12±2.12	u.d.l.	u.d.l.	u.d.l.	0.09±0.01	0.10±0.03	0.78±0.33	0.02±0.01	0.05±0.02
**3MeOBA9THPP**	1.46±0.23	1.22±0.16	2.51±0.32	**55.74±1.23**	0.32±0.03	u.d.l.	0.07±0.02	0.12±0.05	0.91±0.25	0.23±0.11	0.27±0.09
**3MeOBA9ClButP**	0.66±0.16	0.44±0.16	0.97±0.51	u.d.l.	u.d.l.	148.28±12.82	0.01±0.01	0.01±0.00	0.13±0.03	0.01±0.00	u.d.l.
**3MeOBAP9G**	u.d.l.	u.d.l.	**190.42±14.26**	u.d.l.	u.d.l.	u.d.l.	u.d.l.	u.d.l.	u.d.l	0.01±0.00	u.d.l.

CK quantitative analyses were done by UPLC/MS. All values are derived from two biological replicates that were determined in at least two technical replicates. Levels of not shown CKs were under the detection limit of the method. **3MeOBA9THPP** – 3-methoxy(-6-benzylamino-9-tetrahydropyran-2-yl)purine; **3MeOBAP** – 3-methoxy(-6-benzylamino)purine; **3MeOBAPR** – 3-methoxy(-6-benzylamino)purine riboside; **3MeOBAP9G** – 3-methoxy(-6-benzylamino-9-glucosyl)purine; **3OHBAP** – 3-hydroxy(-6-benzylamino)purine; **3OHBAPR** – 3-hydroxy(-6-benzylamino)purine riboside; **3OHBAP9G** – 3-hydroxy(-6-benzylamino-9-glucosyl)purine; **(OG)3OHBAP** – 3-*O*-glucosyl-6-benzylaminopurine; **3MeOBA9ClButP** – 3-methoxy(-6-benzylamino-9-chlorobutyl)purine; **unknown** – 3MeOBA9THPP with additional oxygen. Absolute concentration of unknown metabolite can be misinterpreted as deuterium labeled standard is not available; u.d.l. – under detection limit.

**Figure 5:**
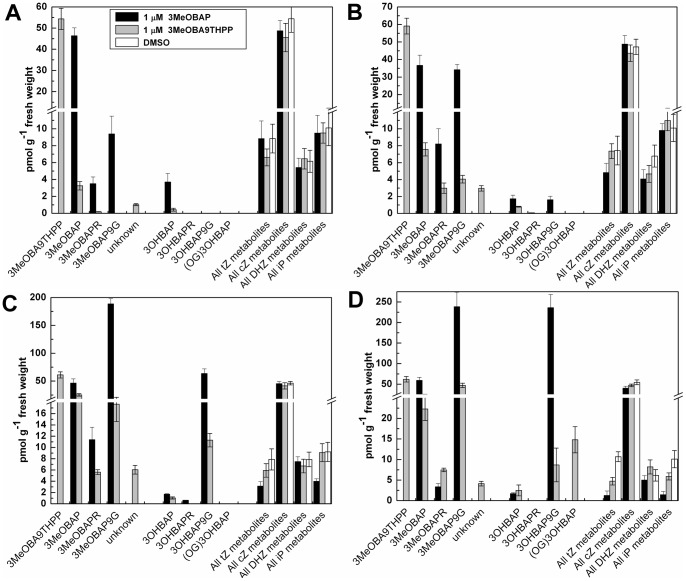
CKs content in maize roots after exogenous application of 1 μM 3MeOBAP and 3MeOBA9THPP compared to controls. CK quantitative analysis was performed by UPLC/MS 1 hour (**A**), 3 hours (**B**), 24 hours (**C**) and 72 hours (**D**) after treatment with 3MeOBAP (black bars), 3MeOBA9THPP (grey bars) and DMSO-treated plants (white bars). All values are derived from three biological replicates that were determined in at least two technical replicates. **3MeOBAPR** – 3-methoxy(-6-benzylamino)purine riboside; 3OHBAP – 3-hydroxy(-6-benzylamino)purine; **3OHBAPR** – 3-hydroxy(-6-benzylamino)purine riboside; **3OHBAP9G** – 3-hydroxy(-6-benzylamino-9-glucosyl)purine; **(OG)3OHBAP** – 3-*O*-glucosyl-6-benzylaminopurine; **unknown** – 3MeOBA9THPP with additional hydroxyl group; **All tZ metabolites** – sum of *trans*-zeatin, its *N9*-riboside, *N9*-glucoside, 5′-monophosphate nucleotide, *O-*glucoside and *N9*-riboside-*O*-glucoside; **All cZ and DHZ metabolites** – sum of above listed *cis*-zeatin and dihydrozeatin derivatives, respectively; **All iP metabolites** – sum of 6*-*isopentenylaminopurine, its *N9*-riboside, *N9*-glucoside and 5′ -monophosphate nucleotide. Absolute concentration of unknown metabolite can be misinterpreted as deuterium labeled standard is not available.

**Figure 6:**
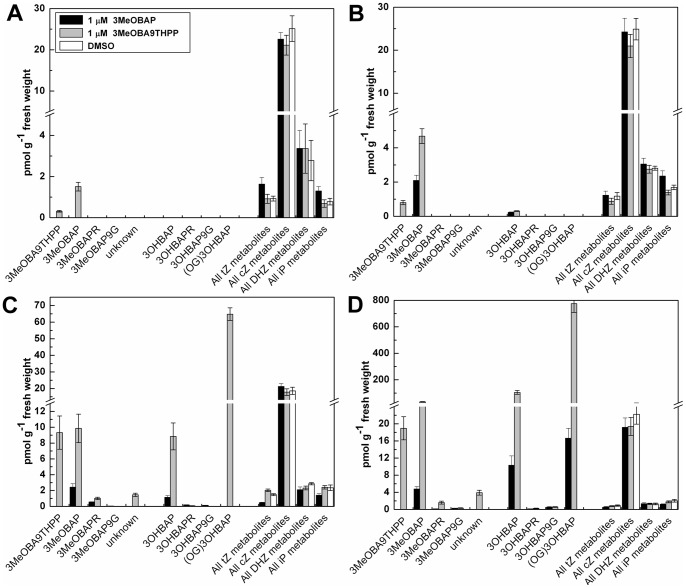
CKs content in maize leaves after exogenous application of 1 μM 3MeOBAP and 3MeOBA9THPP compared to controls. Samples were processed in the same way as the samples from root tissue.

Contrary to feeding with the THP derivative, the major metabolite, which cumulated in the root tissue after free base treatment, was its *N9*-glucoside. *N9*-glucosides of both 3MeOBAP and 3OHBAP comprised the absolute majority (89%) of the total CK content in roots after 3 days (Figure 5). On the other hand, it makes only 33% of the total CK pool after the same period of time in plants treated with the THP-derivative. *N9*-ribosylation proved to be only a minor metabolic conversion and CK nucleotides were under the limit of detection in all cases. Release and accumulation of 3OHBAP in the root tissue was significantly faster when 3MeOBAP was applied. Surprisingly, the total CK contents in leaves of plants treated with THP-derivative increased 30-fold compared to free base treatments, which well corresponded with much a higher content of active 3OHBAP and its *O-*glucoside (Figure 6).

### Content of endogenous isoprenoid cytokinins in plants treated with exogenously applied aromatic cytokinins

Developing maize seedlings predominantly contain isoprenoid types of CKs with a prevalence of *cis-*zeatin derivatives (Figure 5, 6). Naturally occurring aromatic types of CKs have never been detected in maize tissues. There was a remarkable decrease in the intracellular levels of tZ and 6-isopentenylaminopurine (iP) type of derivatives in roots after the application of 3MeOBAP. The decrease was obvious as early as 3 hours after the application reaching approximately 10-fold lower levels after 3 days of treatment compared to controls. However, the application of 3MeOBA9THPP did not have such a strong effect on depletion of endogenous isoprenoid-type of CKs. The levels of *cis*-zeatin metabolites as well as dihydrozeatin types were not significantly influenced in either treatment. The content of isoprenoid CKs in leaf tissue was generally lower and not reduced as distinctively as in roots. Considering levels of tZ and iP types, they decrease to 60% and 87% of their original concentration after the application of 3MeOBAP and 3MeOBA9THPP, respectively.

### Effects of cytokinin application on gene expression profiling

**Figure 7:**
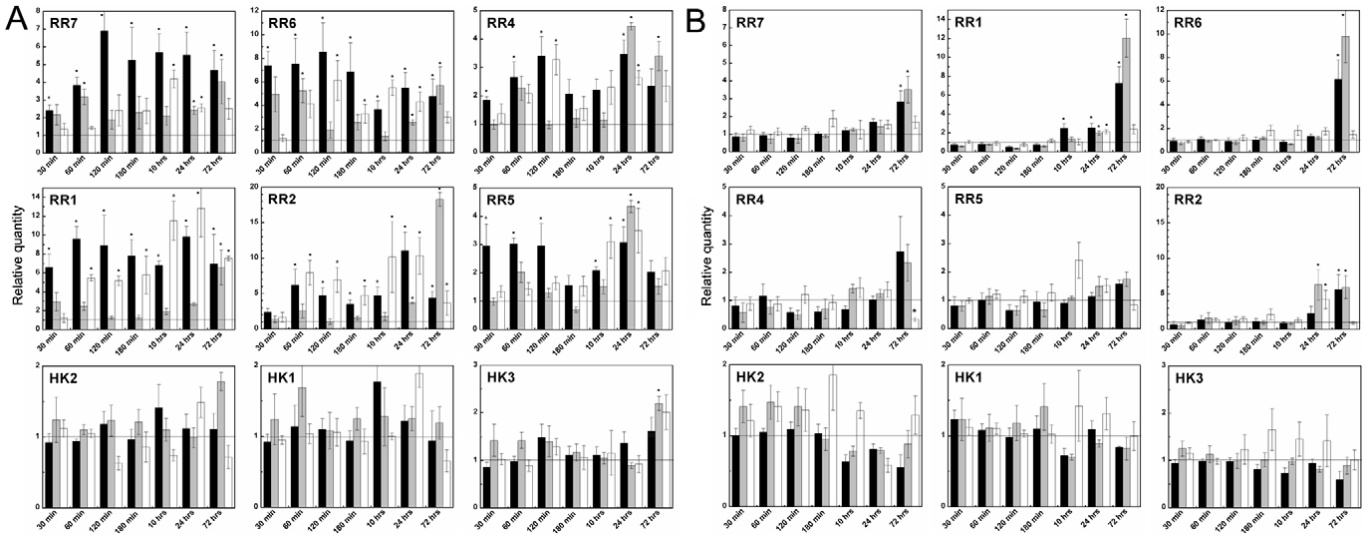
Expression profiles of genes involved in the CK perception and signal transduction after exogenous CK treatments in maize. Changes in expression were followed by qPCR in time intervals ranging from 30 minutes to 3 days in roots (**A**) and aerial part (**B**) of maize plantlets (6 to 8 days of development). **3MeOBAP** (black bars), **3MeOBA9THPP** (grey bars) and **3MeOBAP9G** (white bars) were applied in 1 μM concentration to the nutrient solution. All data are accomplished from three independent biological replicates run in at least two technical replicates. Genes for actin and elongation factor 1 were used as reference genes. Expression change due to control plants considered as statistically significant is indicated by asterisks (unpaired Student's *t* test with *P* ≤ 0.05). **HK** – CK receptor; **RR** – CK response regulator type A. Graphs for *RR* genes are arrayed from left side of upper line to right side of the middle line as abundance of particular gene decreases in given tissue. Graphs for HK genes are ordered from left to right due to their abundance.

Expression of genes involved in CK and ethylene metabolism and CK perception was followed in maize seedlings grown in two concentrations of studied compounds. One-micromolar concentration of 3MeOBAP, its THP derivative and *N9*-glucoside were able to induce expression of CK primary response genes (type A-response regulators; RR) in the roots. 3MeOBAP was able to induce expression already in the first 30 minutes after the application. In contrast, response of THP derivative was little delayed as well as response of *N9*-glucoside (Figure 7). The highest levels of RR expression were observed two hours after the application and then declined modestly, but stayed still significantly up-regulated 3 days after the treatment. The first accumulation of *RR* transcripts was strongest when 3MeOBAP was applied; however, it became comparable after a longer period of treatment (1–3 days) for all three compounds. Contrary to the effect in roots, in leaves we could observe a stronger effect on *RR* transcript accumulation when THP derivative was applied in micromolar concentrations. When 8 nM 3MeOBA9THPP was applied, the transcript levels of *RR* genes were almost unchanged in roots; the free base and 4-chlorobutyl substituted CK up-regulated RR expression even in such a low concentration. In leaves, RR transcript levels were even slightly reduced after one day of nanomolar CK application (Figure S5). The transcript levels for CK receptors were not significantly influenced in comparison with untreated plants, with the exception of low CK concentration, which induced 2 to 3-fold expression of ZmHK2 and ZmHK3 in leaves.

**Figure 8:**
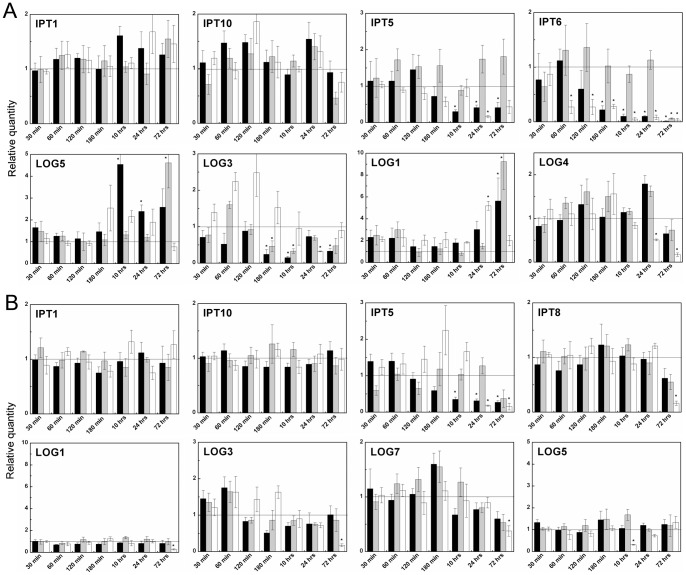
Expression profiles of genes involved in CK biosynthesis and activation after exogenous CK treatments in maize. Changes in expression were followed by qPCR in time intervals ranging from 30 minutes to 3 days in roots (**A**) and aerial part (**B**) of maize plantlets (6 to 8 days of development). **3MeOBAP** (black bars), **3MeOBA9THPP** (grey bars) and **3MeOBAP9G** (white bars) were applied in 1 μM concentration to the nutrient solution. All data are accomplished from three independent biological replicates run in at least two technical replicates. Genes for actin and elongation factor 1 were used as reference genes. Expression change due to control plants considered as statistically significant is indicated by asterisks (unpaired Student's *t* test with *P* ≤ 0.05). **IPT** – isopentenyl transferase (IPT1, 10 – tRNA::IPT, IPT5, 6, 8 – *de novo* IPT); **LOG** – CK nucleoside 5-monophosphate phosphoribohydrolase. Graphs for both groups of genes are arrayed from left to right side as abundance of particular gene decreases in given tissue.

Exogenously applied CKs significantly down-regulated the two most abundant isopentenyl transferases (IPT5 and 6), CK *de novo* biosynthetic enzymes, in both used concentrations (Figure 8, S4). While 3MeOBAP and *N9*-glucoside significantly reduced expression of IPT5 and 6 just three hours after the treatment, 3MeOBAP9THPP did not down-regulate the genes till the third day of the treatment. Gene profiling of CK activating enzymes (LOGs) after treatment did not show straight-forward regulation. The two most abundant genes in roots, *LOG3* and *LOG5*, showed antagonistic pattern of transcript accumulation after 1 μM application. The LOG expression was not significantly affected by 8 nM concentration. The transcripts for two *tRNA::IPTs* (*IPT1* and *10*) were not altered by any applied concentrations.

**Figure 9:**
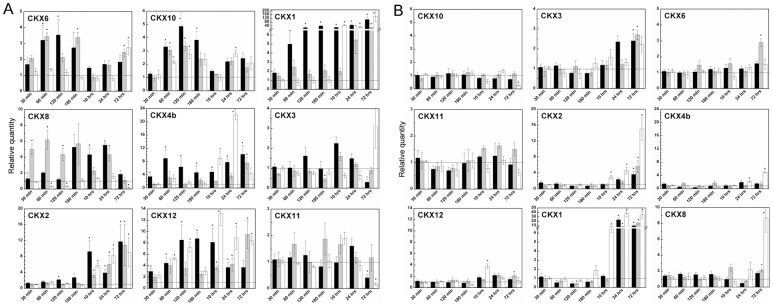
Expression profiles of *CKX* genes involved in CK degradation after exogenous CK treatments in maize. Changes in expression were followed by qPCR in time intervals ranging from 30 minutes to 3 days in roots **(A)** and aerial part **(B)** of maize plantlets (6 to 8 days of development). **3MeOBAP** (black bars), **3MeOBA9THPP** (grey bars) and **3MeOBAP9G** (white bars) were applied in 1 μM concentration to the nutrient solution. All data are accomplished from three independent biological replicates run in at least two technical replicates. Genes for actin and elongation factor 1 were used as reference genes. Expression change due to control plants considered as statistically significant is indicated by asterisks (unpaired Student's *t* test with *P* ≤ 0.05). Graphs are arrayed from left side of upper line to right side of the lower line as abundance of particular gene decreases in given tissue.

Transcripts of cytokinin dehydrogenases (CKXs), CK irreversibly degrading enzymes, accumulated in root tissues in a biphasic way, shortly after application (60 min) and then in long-term response (Figure 9). The two most abundant genes, *CKX6* and *CKX10*, were up-regulated in the short-term response with decreasing intensity from free base, to THP-derivative and *N9*-glucoside. Expression of *CKX1*, the most studied CKX so far with localization in vascular bundles, rose in the time course of CK application. The effect of both *N9*-substituted derivatives was delayed compared to the free base. Other less abundant *CKX* transcripts showed a similar pattern of expression with the exception of *CKX3* and *CKX11* which seemed to not have been affected. Regarding *CKX* transcript levels in leaves, the overall intensity of up-regulation were lower than in roots. Surprisingly, an 8 nM concentration had only a minimal induction effect on the *CKX1* levels compared to a higher concentration, but expression of cytosolic *CKX10* was evidently more up-regulated by lower than by higher CK concentration. An antagonistic pattern of regulation by two order of magnitude different concentrations was observed in several genes e.g. *CKX3*, *IPT7*, *IPT8*, *LOG7*.

**Figure 10:**
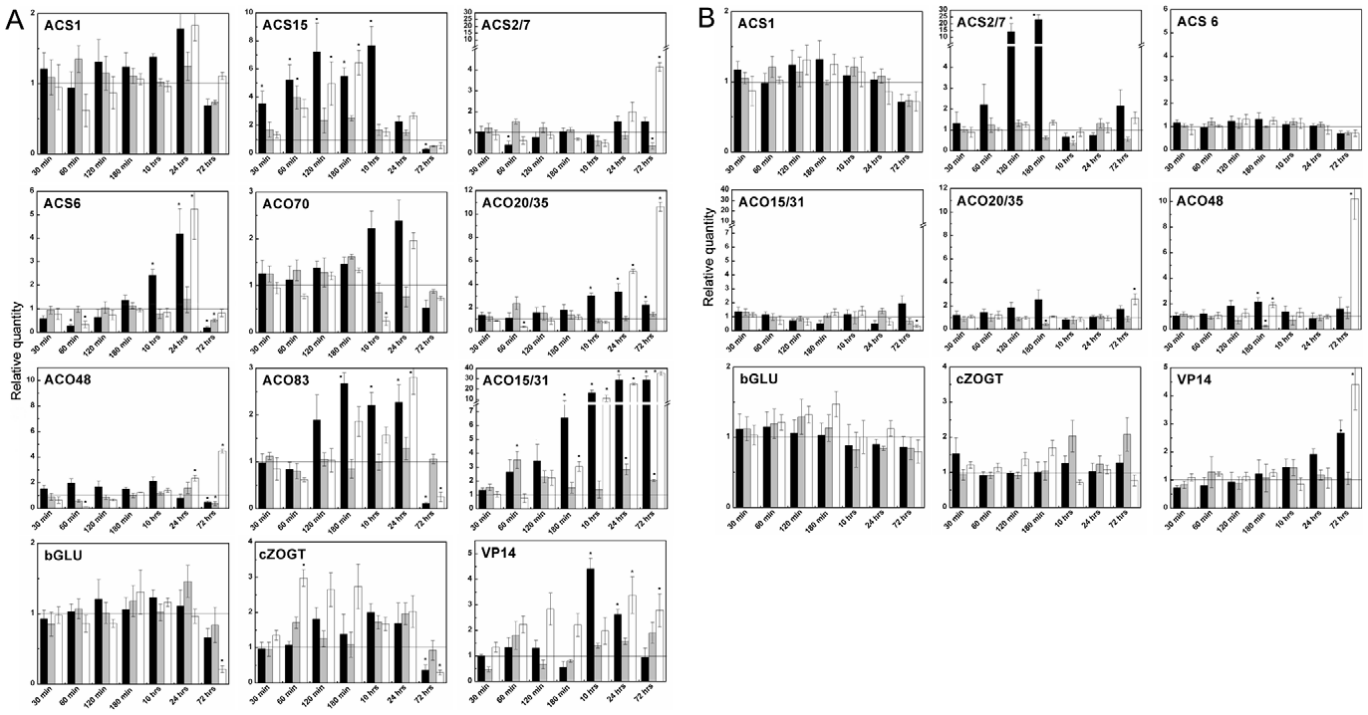
Expression profiles of genes involved in ethylene and ABA production and CK glycosylation and deglycosylation. Changes in expression were followed by qPCR in time intervals ranging from 30 minutes to 3 days in roots (**A**) and aerial part (**B**) of maize plantlets (6 to 8 days of development). **3MeOBAP** (black bars), **3MeOBA9THPP** (grey bars) and **3MeOBAP9G** (white bars) were applied in 1 μM concentration to the nutrient solution. All data are accomplished from three independent biological replicates run in at least two technical replicates. Genes for actin and elongation factor 1 were used as reference genes. Expression change due to control plants considered as statistically significant is indicated by asterisks (unpaired Student's *t* test with *P* ≤ 0.05). **ACS** – ethylene precursor synthase; **ACO** – ethylene release enzyme; **cZOGT** – cis-zeatin-*O*-glucosyltransferase; **bGLU** – *β*-glucosidase; **VP-14** – ABA biosynthetic gene. Graphs for *ACS* and *ACO* genes are arrayed from left to right side as abundance of particular gene decreases in given tissue.

Several genes for 1-aminocyclopropane-1-carboxylic acid synthase (ACS) and oxidase (ACO), ethylene biosynthetic enzymes, had previously been identified [28]. Aside from these, we identified other abundant members of ACS and ACO families in maize genome (Table S2). The application of 3MeOBAP immediately up-regulated expression of the abundant ACS gene (*ACS15*) and after several hours also a majority of ACO and other ACS genes in the roots (Figure 10). Similarly, but a little delayed effect was observed after application of 3MeOBAP9G. The application of 3MeOBA9THPP did not have a significant long-lasting effect on genes involved in ethylene biosynthesis.

### Cytokinin content and CKX activity in xylem sap

Xylem exudates were collected in 30 minutes to 16 hours intervals after the application of 3MeOBAP and its THP derivative to maize roots (Table 2). Two hours after the application of 3MeOBA9THPP, this CK was detected in xylem sap together with its oxidized metabolite in concentration 44.30 and 0.23 nmol μl^−1^ of exudates, respectively. After several hours, the concentration of both compounds significantly increased. The plants treated with the 3MeOBAP had approximately a 10 times lower content of CKs in the xylem sap.

It has never been shown before that CKX protein can be transported via xylem. Surprisingly, xylem exudates only exhibited CKX activity when plants were treated with CKs, whereas the activity was not detectable in DMSO-treated plants. 16 hours after the treatment, plants exposed to free 3MeOBAP base showed a 7 times higher activity (8.9 pkat mg^−1^ of protein) than plants exposed to 3MeOBA9THPP. Since the xylem exudates were collected directly from the base of mesocotyl and specific CKX activity in the aerial part was significantly lower (0.14 pkat mg^−1^), CKX protein detected in xylem sap was most probably root-born.

**Table 2:** Content of CK metabolites in xylem sap of maize seedlings treated with 1 μM 3MeOBAP a 1 μM 3MeOBA9THPP.

Applied CK	Collection time	3MeOBAP	3MeOBAPR	3MeOBAP9G	3MeOBA9THPP	unknown	Total CKs
		nmol in μl of sap					
3MeOBAP	0.5 – 1.0 h	22.50±7.43	0.39±0.09	0.02±0.01	u.d.l.	u.d.l.	22.91
3MeOBA9THPP		10.75±3.43	0.23±0.14	0.11±0.03	21.5±5.92	0.39±0.09	32.98
3MeOBAP	2 – 3 h	8.47±3.02	0.06±0.02	0.40±0.13	u.d.l.	u.d.l.	8.93
3MeOBA9THPP		13.87±4.06	u.d.l.	0.36±0.13	44.3±8.53	0.23±0.10	58.76
3MeOBAP	16 h	11.89±2.87	0.25±0.05	0.56±0.21	u.d.l.	u.d.l.	12.70
3MeOBA9THPP		8.86±1.92	u.d.l.	0.12±0.05	76.00±12.62	10.18±1.76	95.16

CK quantitative analysis was carried out by UPLC/MS in approximately 30 μl of collected sap 0.5 to 1 h, 2 to 3 h or 16 h after CK application. All values are derived from two biological replicates that were determined in at least two technical replicates. Concentration of all other CKs in xylem exudates were under the detection limit of the method; u.d.l. – under detection limit.

## Discussion

The main intention of the project was to study the effect of various CK analogues on plant morphology and describe molecular aspects of their application. First, we tested suicide inhibitor of CKX – *N^6^*-pent-2,3-dienyl-aminopurine, whose application led to the retention of elevated levels of endogenous CKs in potato [29] and strong competitive inhibitor N-(2-amino-pyridin-4-yl)-N'-phenylurea of the same enzyme [30]. Nevertheless, both compounds had a strong inhibitory effect on root elongation and branching (Figure S1). They strongly up-regulate *RR* and *CKX* genes immediately after application in a similar way as free active CK bases. The effect was even more cumulative as they cannot be effectively deactivated in plant tissues (data not shown). *N9*-THP derivative of 3MeOBAP was the only compound later selected showing comparable activities to classical CKs in the standardized CK tests without negative effect on root growth.

Exogenously applied CK with free hydroxyl group yields to extensive *O-*glucosylation. Hence, methoxy-group can protect this rapid metabolic conversion and decrease accumulation of *O-*glucosides in certain tissues. Equally, the *N9*-THP group protects CK molecule against *N9*-glycosylation. Although metabolic study with radioactively labeled 3MeOBA9THPP revealed that both groups can be slowly eliminated *in vivo*, the total content of inactive glucosides is still significantly lower in comparison to treatments with the unprotected molecule. Nevertheless, the *in vivo* elimination of protective groups is most likely crucial for consideration of such a derivative as an active CK. Standardized CK bioassays usually do not show real CK features of the tested compounds but the potency of its metabolic products. *N-* and *O-*glucosides were found to be unable to trigger CK receptors [12], [31]. Accordingly, none of our tested *N9*-substituted 3MeOBAP derivatives were able to activate maize or Arabidopsis receptors. 3MeOBAP has never been tested to trigger receptors themselves; despite the observation that the compound, together with its riboside, is very potent in various CK bioassay [32]. A bacterial test on CRE1/AHK4 receptor, which is predominantly expressed in Arabidopsis roots [33], revealed that 3MeOBAP is unable to activate the transduction pathway up to 50 μM concentration. Accordingly, structural resolution of the AHK4 receptor with different ligands revealed that all three nitrogen atoms (*N3*, *N7*, *N9*) of cytokinin purine ring are buried in the binding pocket of the receptor and thus cytokinins substituted on these positions could not efficiently induce CK-signalling pathway [34]. Activation of the other tested receptors in the presence of 3MeOBAP was detected; however, in much higher concentrations in comparison with 3OHBAP or BAP (Figure 4). Hence, it can be assumed that 3MeOBA9THPP and released 3MeOBAP will not significantly contribute to CK sensing *in planta*. CK responsiveness in tissues will be dependent mostly on the effective concentration of de-protected free CK bases (in our case 3OHBAP). This is in accordance with our observations of activation of CK primary response genes in maize seedlings. These genes became strongly up-regulated when 3MeOBAP was applied in the first hours after the compound uptake by the roots. Content of the active 3OHBAP is almost one order of magnitude higher 1 hour after the treatment contrary to the treatment with 3MeOBA9THPP. Nonetheless, later when 3OHBAP is released in similar concentrations also from 3MeOBA9THPP and 3MeOBAP9G, the RR transcript levels are becoming more proportional. On the other hand, a higher content of 3OHBAP in the leaves of plants treated with 3MeOBA9THPP reflects upper transcript levels of *RR* genes compared to plants treated with 3MeOBAP. The discrepancy in the contents of active 3OHBAP in plants treated with CK with or without THP substituent is due to significantly better transport of THP derivative via xylem into the aerial part of plant and subsequent metabolic conversion by demethylation which is more prevalent in leaves. *N9*-ribosides have been hypothesized previously to be major acropetally transported forms of endogenous CKs [13], [35]. Accordingly 3MeOBAP riboside was detected in xylem sap in relatively high concentrations contrary to *N9*-glucosides; however 3MeOBA9THPP seems to be transported even more efficiently as apparent from their proportional representation between root tissue and xylem sap.

Up-regulation of CK degradation genes together with down-regulation of biosynthetic genes after CK exogenous application results in a significant decrease in isoprenoid CK levels which are the preferred substrates and products of the above metabolic steps. When plants were treated with *N9*-unprotected CK approximately 90% depletion of tZ and iP forms was observed 72 hours after application. Since any down-regulation of *RR* genes was not observed, 3OHBAP originated from its corresponding derivative probably supplies the CK sensing role. Longer treatment with 3MeOBAP and 3MeOBAP9G stimulates expression of ethylene biosynthesis genes more intensively than the application of 3MeOBA9THPP. Activation of ethylene biosynthesis is thus nicely correlated with observed root phenotypes and inhibited root cell elongation. Conversely, thickening of the root body can be attributed to disruption of asymmetric distribution of CK and auxin levels between cortical parenchyma and stele described recently on maize root seedlings [36]. A massive uptake of CK by roots and its centripetal flow into the stele increases its local concentration in tissue with predominant auxin accumulation and thus, probably accelerates cell division in naturally non-dividing cell layers. Similarly, augmented CK concentration in the root caused by the exogenous application counteract the auxin promotive action on lateral root formation [37] as the thickening is accompanied by reduction of lateral root number.

Our recent work [38] shows that exogenously applied kinetin derivatives substituted at *N9*-position with similar protective groups as we used in this study, have an impact on antisenescence potency as well as stress acclimation. Endogenously applied CKs greatly activate production of ABA, considered as a stress hormone in maize leaves [39]. Both 3MeOBAP and its *N9*-glucoside up-regulate expression of ABA biosynthetic gene (*VP14*) more clearly than 3MeOBA9THPP. THP protection can thus attenuate stress impact of applied CK and better sustain growth and productivity of plants, which we indeed observed.

Precise CK quantifications in maize seedlings after the application of different 3MeOBAP derivatives showed that the protective effect of *N9*-chlorobutyl group is not sufficient as it leads to an increase of the total uptake of CK by the root and an even higher amount of active CK is released compared to the treatment with free base itself. In a similar fashion both compounds (3MeOBAP and 3MeOBA9ClButP) up-regulated the *RR* genes even in 8 nM applied concentration (Figure S5). However, the quantification does not clearly explain a similar negative effect of 3MeOBAP9G on root growth as application of the free base instead of simulating the effect of 3MeOBA9THPP. The significant difference in transport of the two compounds should be taken into consideration. Another hypothesis might arise from the fact, that treated tissues accumulated an enormous quantity of non-active CK derivatives, although they are not able to trigger CK receptors, they could in a higher concentration competitively inhibit their active sites and thus act as so called anti-cytokinins. Recently, two structures derived from hydroxylated aromatic CKs have been described to effectively compete with tZ in binding tests with two Arabidopsis CK receptors and repress induction of CK response genes [40], [41]. 6-(2-hydroxy-3-methylbenzylamino)purine and 6-(2,5-dihydroxybenzylamino)purine can structurally resemble an unknown derivative, which originates in roots treated with 3MeOBA9THPP and after 72 h forms almost 40% of the initial amount of applied compound detected in leaves (based on conversion of radioactively labeled standard; Table S1). A mass detection analysis revealed that the unknown compound is in fact original 3MeOBA9THPP with additional hydroxyl group. The gene expression profiling after 8 nM application of the CKs shows that some of the *RR* genes can be down-regulated and thus support the anti-CK effect of the compound. Hence, the positive effect of applied *N9*-substituted 3MeOBAP on root proliferation and lateral branching, observed in nanomolar applications, can result from the inhibitory effect of originated hydroxy metabolite on CK receptors, which cannot be exceeded by slow and insufficiently released active 3OHBAP from its precursor. Observed root phenotypes matched to these of plants fed with micromolar dosage of the above mentioned anti-cytokinins [40], plants with knocked-out receptors [42] or plants with depleted content of endogenous CKs by overexpression of CKX genes [43].

Based on the precise metabolite quantification, the most intriguing data reveal differences in *in vivo* conversions between the two used model plants. For the first time, we have shown that some plant tissues are able to revert the inactive *N9*-glucosides back to its active forms. Based on previous studies mostly carried out on radish, *N9*-glucosides had always been regarded as terminal metabolic products [44], [45]. The premise arose also from the *N9*-glucosides structural feature with resonance delocalization of the free electrons of the anomeric nitrogen atom [46] and from low or zero activities detected in bioassays which are in fact usually performed on very specific tissues such as calli, and can hold limited metabolic machinery. Whereas Arabidopsis seedlings indeed do not possess deglycosylation enzymes, able to hydrolyze glucosyl residue bound to *N9*-position of CK [14], such a glucosidase is probably activated in young maize roots as we monitored a release of deglycosylated CK in a fairly significant quantity. It was previously shown that *N9*-THP derivatives can be hydrolyzed non-enzymatically in the acidic pH [1] to which can lower the environment of lytic vacuoles. However, we did not confirm that 3MeOBAP9G or 3MeOBA9ClButP could yield *in vitro* into some instability down to pH 4 (Table S3).

Actually, the only plant *β-*glucosidase, with specificity toward CK glucosides that was characterized up to now is maize Zm.p60-1 which preferentially deglycosylates *N3*- and *O-*glucosides [47]. Zm.p60.1 belongs to the large group of hydrolases family GH1, based on amino acid sequence and protein ternary structure, counting for instance 48 and 40 members in Arabidopsis and rice genomes, respectively. Most of these GH1 *β-*glucosidases are closely related to one another but Zm.p60.1 does not cluster with any of the Arabidopsis or rice GH1 clades [48]. Hence, the plastid targeted Zm.p60.1 and its closely related orthologues found in wheat, barley and other cereals probably do not have functional counterparts in rice and Arabidopsis and the manner of CK deglucosylation can consequently vary in these species. Recently, recombinant Zm-p60.1 was shown to hydrolyze tZ-*N9*-glucoside *in vitro*, albeit at a very low reaction rate [49]. However, it is still rather speculative if the *β-*glucosidases can contribute to activation of cytokinin *N9*- glucosides *in vivo* in untreated plants or the observed hydrolysis of cytokinin *N9-*glucosides is only consequence of enormous increase of enzymés substrate after exogenous application in cellular compartments with its predominant localization.

In conclusion, we have shown that proper combination of alkyl substituents on CK molecule can lead to a gradual conversion to free active base and increased transport rates in plant vasculature, resulting in a generally positive effect on plant vitality without root inhibition phenotype usually observed after exogenous CK applications. For this reason, and also because of relatively low costs of their production, 3MeOBA9THPP or 3MeOBA9ClButP appear to be a suitable alternative to BAP and other CKs in various CK applications in micropropagations etc. 3MeOBA9THPP was already tested for micropropagation of several *Ulmus* species and in contrast to commercially used BAP it did not show negative effect on root formation and proliferation while positive effect on shoot development was obvious and comparable with the effect of BAP (Doležal and Malá, manuscript in preparation). Testing of 3MeOBA9THPP and related analogues in other micropropagation plant systems is currently in progress in our and collaborating laboratories. However, variability in metabolic machinery among various plant species needs to be considered in every particular application as well as the effective concentration.

## Materials and Methods

### Plant material and phenotype characterization

*Arabidopsis thaliana* seeds (ecotype Columbia) were sterilized with 70% ethanol and 0.1% Triton X-100 and then washed with sterile water. The seeds were transferred to vertical square petri dishes on a solid medium consisting of MS (2.15 g l^−1^), MES (0.5 g l^−1^), sucrose (1 g l^−1^), agar (11 g l^−1^), buffered to pH 5.8, and various concentrations of CKs. The compounds were dissolved in DMSO, thus 0.05% DMSO was used as a control solution. After incubation for 3 days in darkness at 4°C, the seeds were germinated and grown in an environmental chamber (fluorescence light intensity 150 μE m^−2^ s^−1^, humidity 55%, 16 h day/8 h night, 22°C). After 7 and 14 days respectively, the length of the primary root was evaluated using Scion Image software (Scion Corporation, Frederick, MD, USA). The number of fully emerged lateral roots was scored under a stereomicroscope. When different metabolites were quantified, seeds (100 per 50 ml) were cultivated in the same liquid MS medium under the same environmental conditions with continuous shaking at 90 rpm for 14 days. CKs were added for the last three days of cultivation. Dry kernels of *Zea mays* cv. CELLUX were imbibed in tap water and germinated on a wetted filter paper. After 3 days, the seedlings were transferred to aerated hydroponic tanks filled with Hoaglands nutrient solution. The plants were grown in an environmental chamber with 16 h light period (250 μmol m^−2^ sec^−1^) at 27°C and 8 h dark period at 20°C. CKs were added to a nutrient solution two days after seedlings were nested in the tanks. Length of primary root and the first leaf were determined with ruler on sets of twelve plants; each treatment was evaluated at least in 10 independent tanks. Dry mass of root system and the aerial part was determined after cutting off the kernel and desiccation in laboratory oven for two days at 60°C. All measured parameters were statistically evaluated between untreated and treated plants according to Student's unpaired t-test.

### Identification and quantification of cytokinin metabolites

[^15^N_4_] 3MeOBA9THPP was prepared by condensation of 6-chloro[^15^N_4_]purine and 3-methoxybenzylamine in n-buthanol in the presence of triethylamine. THP substituent was added to *N9* position by procedure described in [1]. Tritium-labeled BAP9G and DHZ9G were obtained from OlChemIm (Olomouc, Czech Republic). Samples from radioactive labeled CK treated plants were extracted in 70% ethanol at −20°C. After 3 hours incubation and re-extraction, pooled supernatants were passed through C18 SPE column and analyzed using the HPLC-UV system (Symmetry C18 column 5μm, 2,1×150 mm; Waters, Milford, MA, USA). Other procedures for CK quantification were based on immunoaffinity purification method [50]. Contrary to published procedure, samples were extracted overnight by 70% ethanol to avoid acidified Bielski buffer, which caused instability of THP derivatives. Deuterium-labeled CKs (OlChemIm) were added as internal standards, each at 1 pmol per sample [51]. The methanolic eluates from immunoaffinity columns were evaporated to dryness and dissolved in 20 µL of the mobile phase. The samples were analyzed by ultra-performance liquid chromatography (Acquity UPLC™; Waters) coupled to a Xevo TQ MS ™ API (Waters) triple quadrupole mass spectrometer equipped with an electro-spray interface. The purified samples were injected onto a C18 reversed-phase column (BEH C18; 1.7 μm; 2.1×50 mm; Waters). The column was eluted with a linear gradient (0 min, 10% B; 0 to 8 min, 50% B; flow-rate of 0.25 mL min^−1^; column temperature of 40°C) of 15 mM ammonium formate (pH 4.0, A) and methanol (B). Quantification was achieved by multiple reaction monitoring of [M+H]^+^ and the appropriate product ion. The quantification was performed by Masslynx software using a standard isotope dilution method.

### Bacterial Cytokinin receptor In-vitro Assay

Arabidopsis receptor activation assay were done as described previously [26], [52]. Full-length coding sequences of maize receptors ZmHK1, ZmHK2 and ZmHK3 were obtained by gene synthesis (GeneArt, Invitrogen) and cloned into pPIN-III. Activity of *β*-galactosidase in *E.coli* strain KMI001 (Δ*rcsC, cps::lacZ*) was measured using 4-methylumbelliferyl β-D-galactopyranoside as a substrate after induction with 25 µM IPTG and overnight growth in the presence of CK as we described previously [12].

### Quantitative PCR Analysis

Expression profiling of genes coding for CKX, IPT, RR and HK and evaluation were done as described previously [39]. Briefly, total RNA isolated using RNAqueous® kit and treated twice with TURBO DNase-free™ kit (Life Technologies, Foster City, CA, USA) was used for first strand cDNA synthesis by RevertAid™ H Minus M-MuLV RT and oligo-dT (Fermentas, Vilnius, Lithuania). Diluted cDNA samples were used as templates in real-time PCR reactions containing POWER SYBR® Green PCR Master mix or TaqMan® Gene Expression Master Mix (Life Technologies) and 300 nM of each primers and 250 nM specific 5′-FAM and 3′-NFQ labeled MGB or standard Taqman probe, respectively. RNA from every biological replicate was transcribed in two independent reactions and each cDNA sample was run in at least two technical replications on StepOnePlus™ Real-Time PCR System in a default program (Life Technologies). C_t_ values were normalized with respect to *β-*actin and elongation factor 1 genes. Expression values were determined and statistically evaluated with DataAssist v3.0 Software (Life Technologies). The Benjamini and Hochberg false discovery rate was used to obtain adjusted p-values for unpaired t-test [53]. The sequences for maize *LOG* were generated from annotated maize genome (http://www.maizesequence.org/index.html) on the basis of homology to rice orthologues [54]. Primers and probes for *LOG*, *ACS* and *ACO* genes (Table S2) were designed the same way. Regarding high homology of some pairs of *ACS* and *ACO* genes (more than 92%), primers were designed to be 100% identical to both sequences and quantification was determined for a mixture of both transcripts.

### Collection of root xylem fluids and measurement of cytokinins and CKX activity

The shoots of maize plantlets were cut off at a distance approximately 0.5 cm from the mesocotyl base. Exudates were collected during following 30 to 60 min from the cut surface of the mesocotyl by micropipette. The first 5 μl of the exudates were discarded to avoid contamination by other saps. The exudates were cooled on ice during the collection period and subsequently frozen at −70°C. Collection of exudates was started in three different time-points (0.5 h, 2 hrs and 16 hrs) after the application of studied compounds into the nutrient solution. For CK quantification, exudates were passed through C18 spin up columns, aliquots diluted in mobile phases (1/10) and applied on UPLC/MS. CKX activity was estimated due to previously described end-point spectrophotometric method where conjugate of reaction product (3-methyl-2-butenal) with 4-aminophenol was determined [6]. Enzymatic reaction was carried out in 100 mM McIlvaine buffer, pH 7.5, with 500 µM 2,6-dichlorophenolindophenol as an electron acceptor and 250 µM iP as a substrate.

## Supporting Information

Figure S1 **Phenotypes of Arabidopsis 14-day-old seedlings grown on MS agar media supplemented with 3MeOBAP derivatives.** **DMSO** – control plants treated with dimetylsulfoxide; **HA-1** – *N^6^*-pent-2,3-dienyl-aminopurine; **ACPU** – N-(2-amino-pyridin-4-yl)-N'-phenylurea.
(TIF)

Figure S2 **Maize phenotypes after exogenous treatments with 3MeOBAP derivatives**. CKs were supplemented to the nutrient solution 4 days after germination; ten seedlings were cultivated in one liter of medium. Photos were taken 7 days after CK treatments.
(TIF)

Figure S3 **The spectra of (OG)3OHBAP (A), 3MeOBAP9G (B), 3MeOBAP + 3MeOBA9THPP+OH (unknown derivative; C), 3OHBAP (D), 3MeOBAP + 3MeOBA9THPP (E) in experiment where plants were fed by 1**∶**1 [^15^N_4_] 3MeOBA9THPP and 3MeOBA9THPP.**
(TIF)

Figure S4 **The retention time of radioactivity detected after application of tritium labeled BAP9G and DHZ9G. (A)** The radioactivity of the standard which was added to the media; **(B)** the radioactivity in the shoot; **(C)** the radioactivity in the root; **(D)** the radioactivity in the media after treatment.
(TIF)

Figure S5 **Expression profiles of genes involved in CK metabolism and perception after exogenous application of 8 nM and 1 μM CKs to maize seedlings.** Changes in expression were followed by qPCR in two time-points in **roots** – 120****minutes after feeding (**A**) and 3 days after feeding (**B**) and two time points in the aerial part – 1****day after feeding (**C**) and 3****days after feeding (**D**). 3MeOBAP, 3MeOBA9THPP and 3MeOBA9ClButP were applied in **1 μM** concentration (**black bars**) or **8 nM** concentration (**light bars**) to the nutrient solution. All data are accomplished from three independent biological replicates run in at least two technical replicates. Expression change due to control plants considered as statistically significant is indicated by asterisks (unpaired Student's *t* test with *P* ≤ 0.05).
(TIF)

Table S1 **The retention time (RT) of radioactivity detected after application of labeled 3MeOBA9THPP.** CK standards eluted in corresponding RTs are listed together with level of detected radioactivity.
(DOC)

Table S2 **Sequences of primers used in qPCR.** Accession numbers of putative proteins from Maizesequence database (http://www.maizesequence.org/index.html). Number of exons and chromosome location are listed together with GeneBank accession numbers and reference if gene has been previously characterized. Transcript abundance is expressed as gene copy number in 1****ng of total RNA amplified in qPCR.
(DOC)

Table S3 **Stability of** ***N9*** **-derivatives in acidic solutions.** The pH stability of compounds in McIlvaine buffer with pHs decreasing from 5.5 to 4 measured 16****h after sample preparation. The percentage of the released free base 3MeOBAP was determined by HPLC.
(DOC)
