# Supplementary figures and images for: Novel Cytokinin Derivatives Do Not Show Negative Effects on Root Growth and Proliferation in Submicromolar Range
Source: PLoS One. 2012 Jun 18;7(6):e39293. doi: 10.1371/journal.pone.0039293 (PMC3377648; doi:10.1371/journal.pone.0039293)

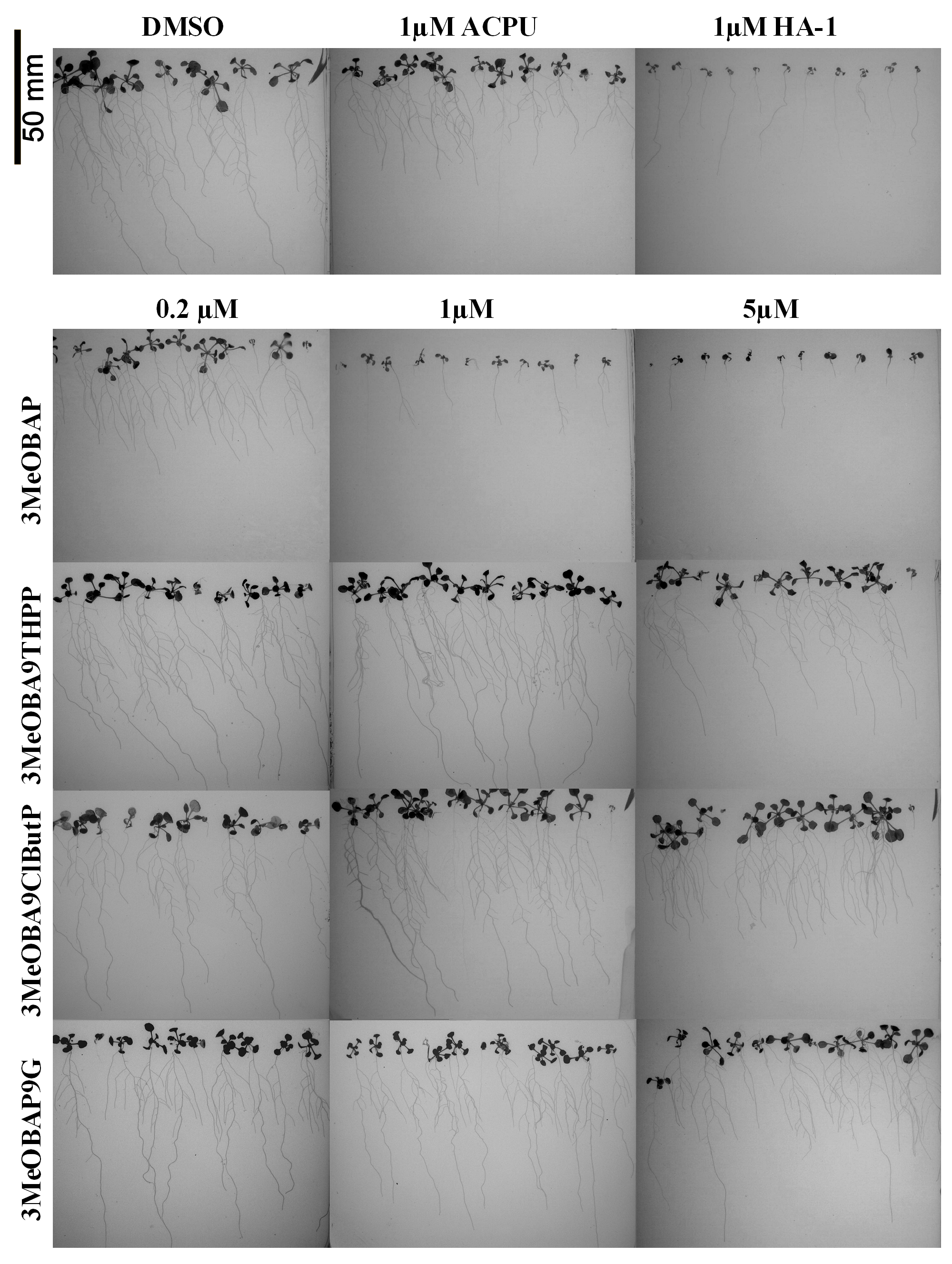

Supplement: Figure S1 — Phenotypes of Arabidopsis 14-day-old seedlings grown on MS agar media supplemented with 3MeOBAP derivatives. DMSO – control plants treated with dimetylsulfoxide; HA-1 – N6-pent-2,3-dienyl-aminopurine; ACPU – N-(2-amino-pyridin-4-yl)-N'-phenylurea. (TIF) [file pone.0039293.s001.tif]

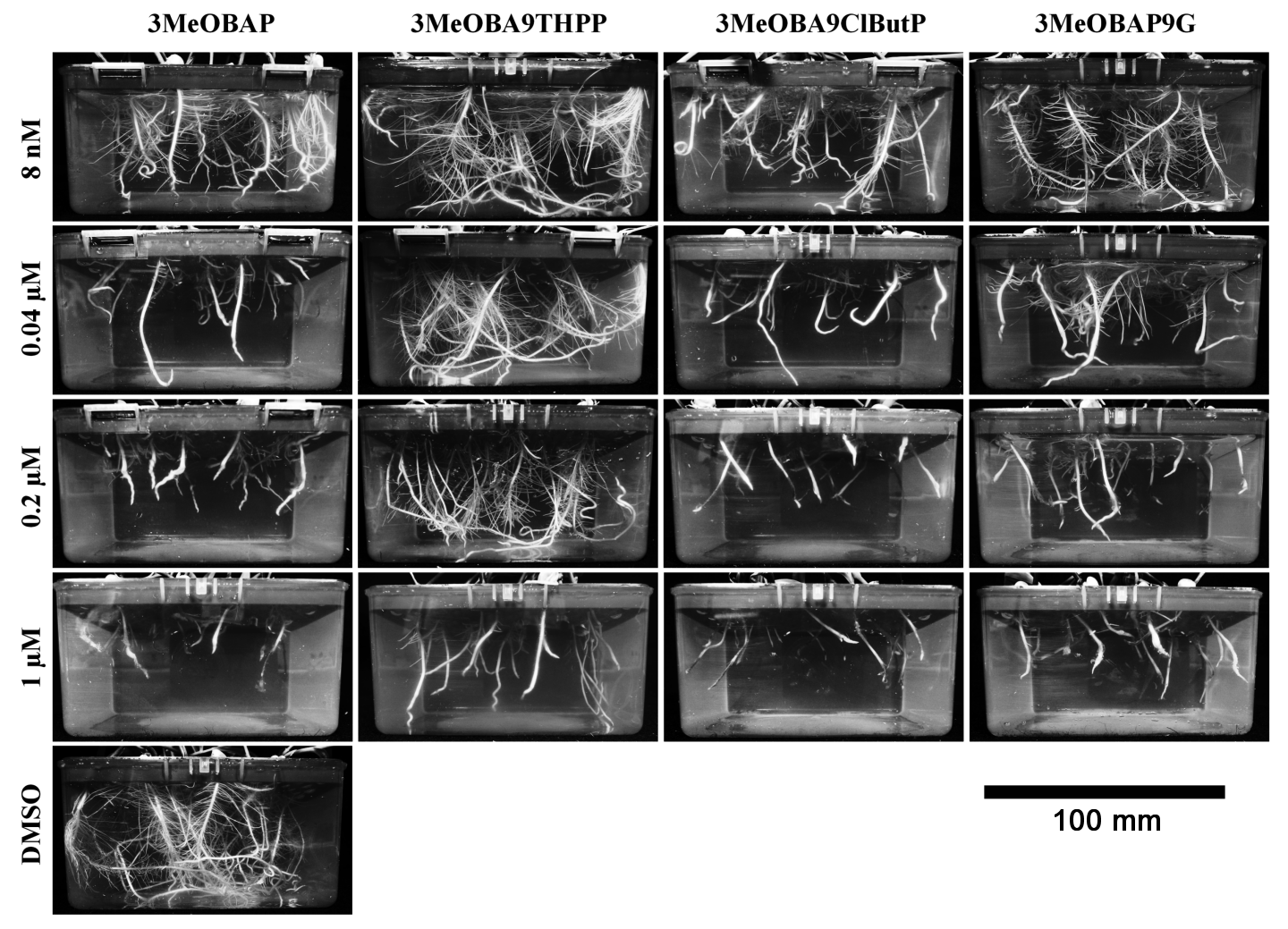

Supplement: Figure S2 — Maize phenotypes after exogenous treatments with 3MeOBAP derivatives. CKs were supplemented to the nutrient solution 4 days after germination; ten seedlings were cultivated in one liter of medium. Photos were taken 7 days after CK treatments. (TIF) [file pone.0039293.s002.tif]

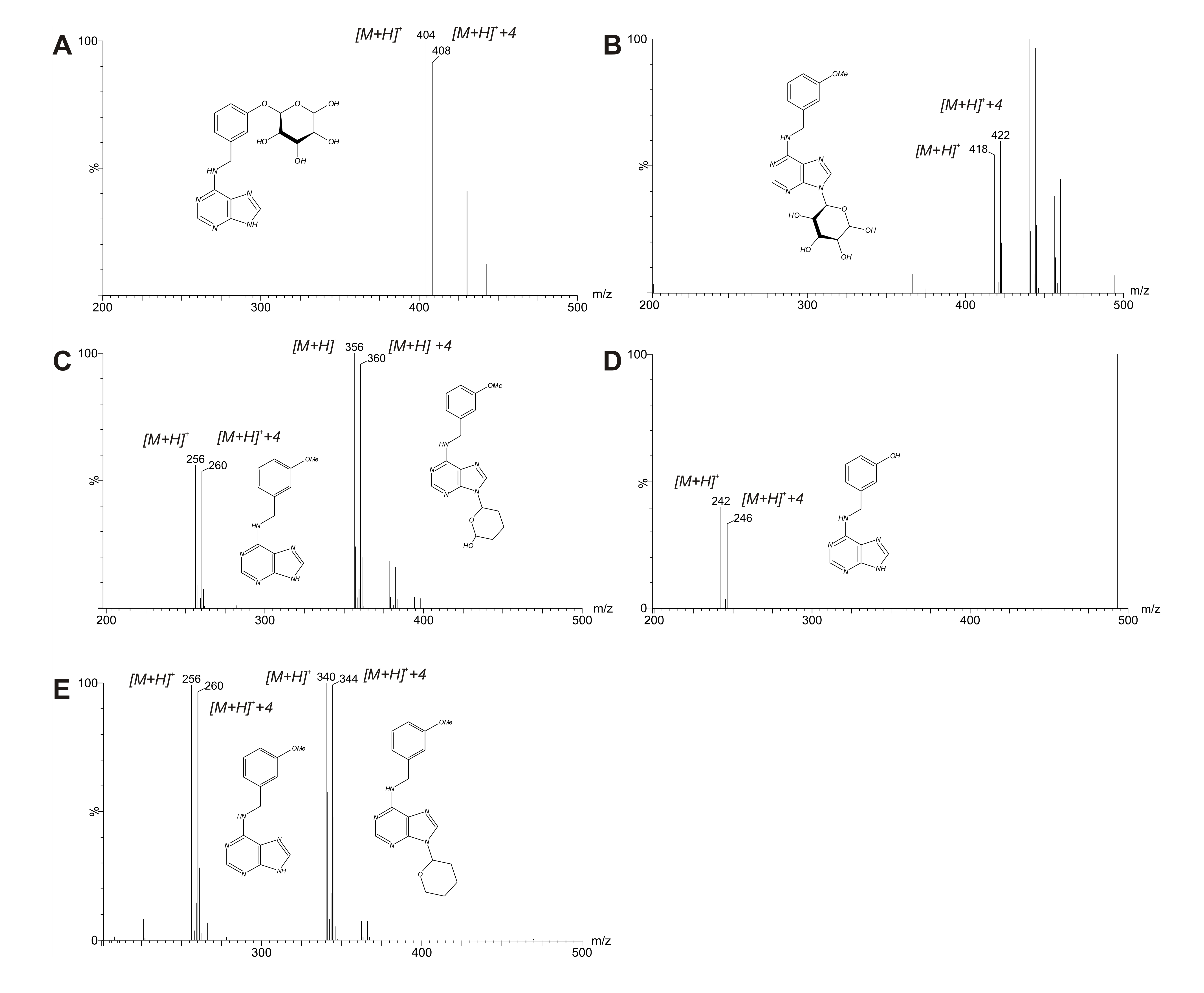

Supplement: Figure S3 — The spectra of (OG)3OHBAP (A), 3MeOBAP9G (B), 3MeOBAP + 3MeOBA9THPP+OH (unknown derivative; C), 3OHBAP (D), 3MeOBAP + 3MeOBA9THPP (E) in experiment where plants were fed by 1∶1 [15N4] 3MeOBA9THPP and 3MeOBA9THPP. (TIF) [file pone.0039293.s003.tif]

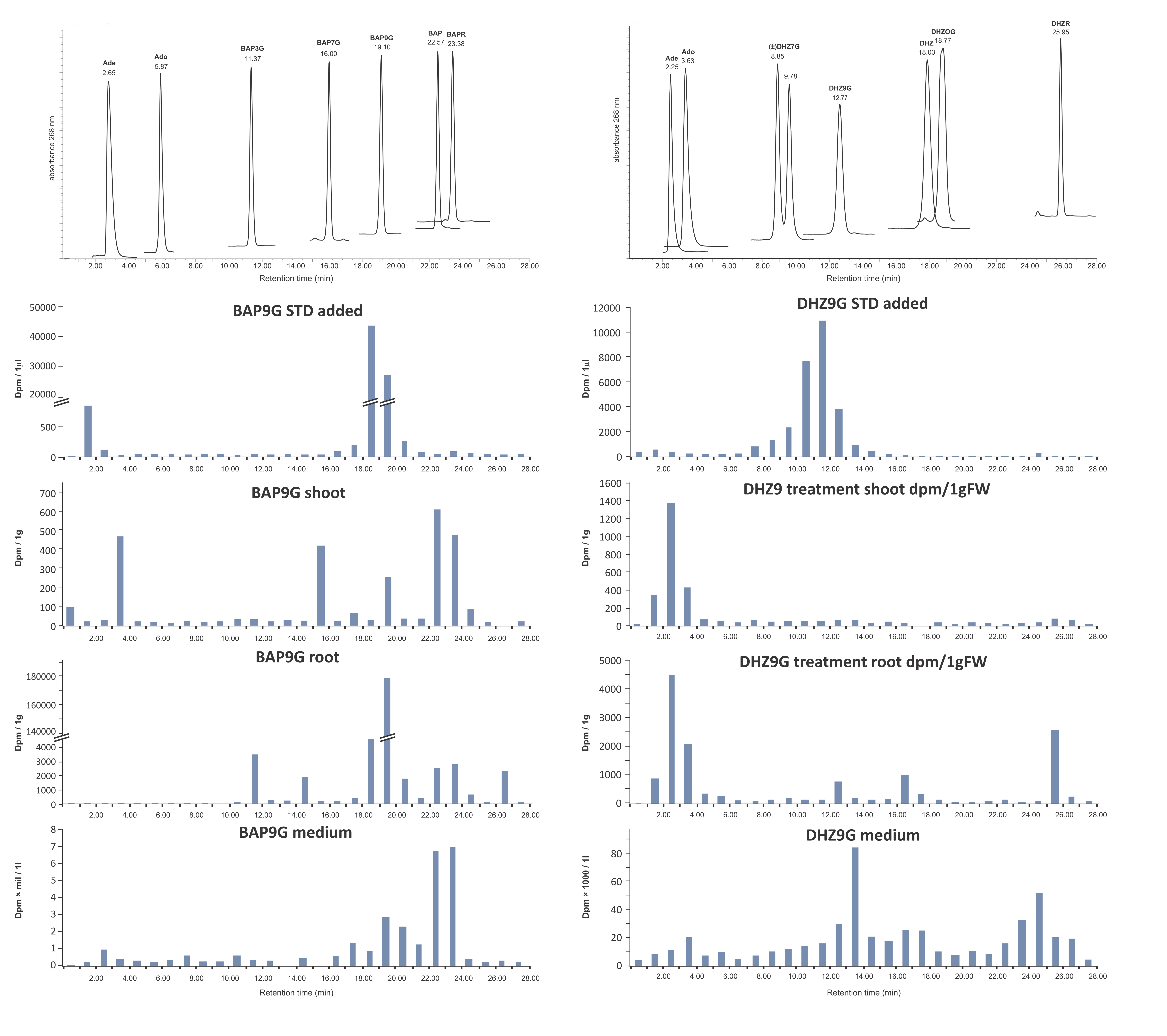

Supplement: Figure S4 — The retention time of radioactivity detected after application of tritium labeled BAP9G and DHZ9G. (A) The radioactivity of the standard which was added to the media; (B) the radioactivity in the shoot; (C) the radioactivity in the root; (D) the radioactivity in the media after treatment. (TIF) [file pone.0039293.s004.tif]

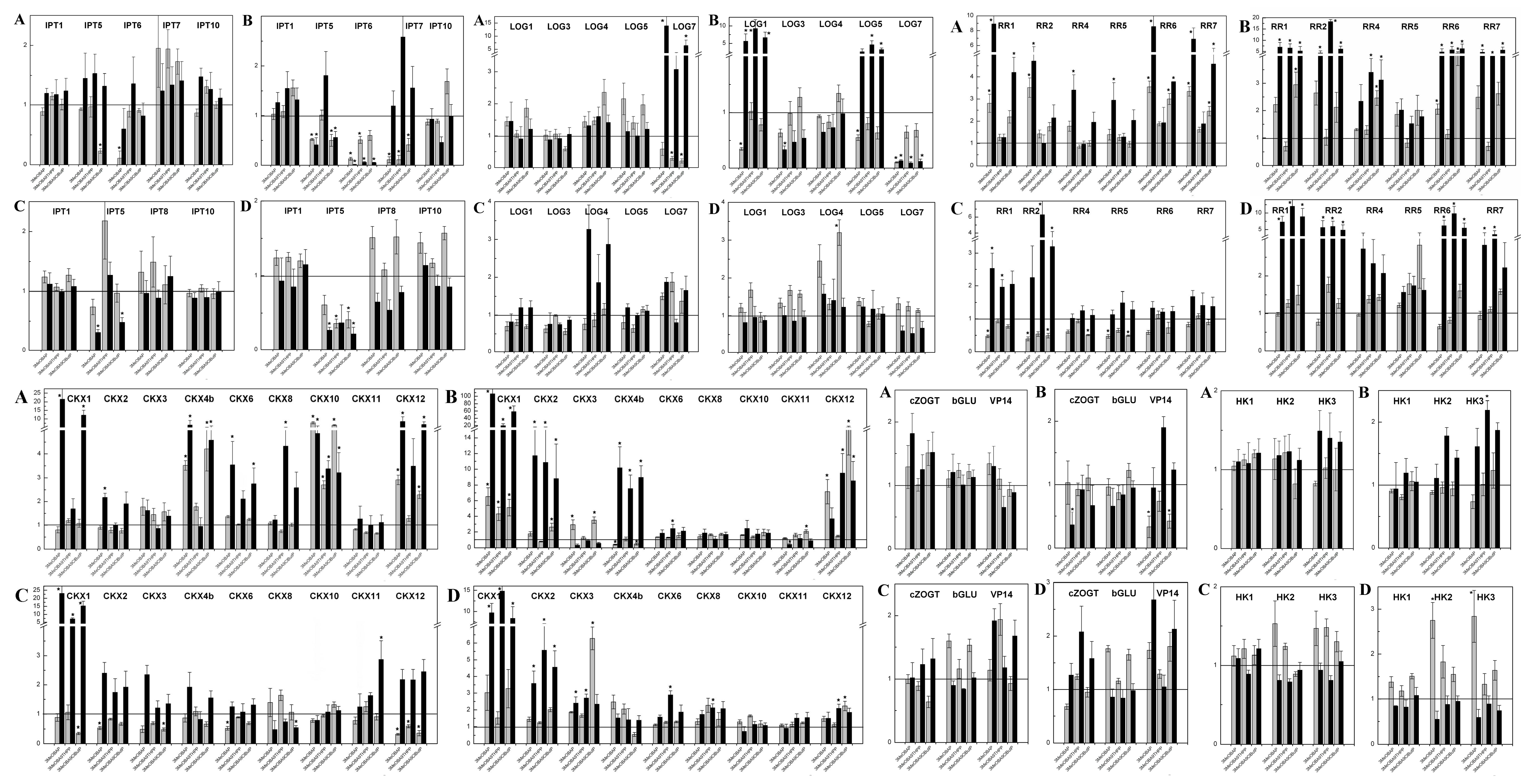

Supplement: Figure S5 — Expression profiles of genes involved in CK metabolism and perception after exogenous application of 8 nM and 1 μM CKs to maize seedlings. Changes in expression were followed by qPCR in two time-points in roots – 120minutes after feeding (A) and 3 days after feeding (B) and two time points in the aerial part – 1day after feeding (C) and 3days after feeding (D). 3MeOBAP, 3MeOBA9THPP and 3MeOBA9ClButP were applied in 1 μM concentration (black bars) or 8 nM concentration (light bars) to the nutrient solution. All data are accomplished from three independent biological replicates run in at least two technical replicates. Expression change due to control plants considered as statistically significant is indicated by asterisks (unpaired Student's t test with P ≤ 0.05). (TIF) [file pone.0039293.s005.tif]
