# Supplementary material for: Novel Cytokinin Derivatives Do Not Show Negative Effects on Root Growth and Proliferation in Submicromolar Range
Source: PLoS One. 2012 Jun 18;7(6):e39293. doi: 10.1371/journal.pone.0039293 (PMC3377648; doi:10.1371/journal.pone.0039293)
Supplement: Table S1 — The retention time (RT) of radioactivity detected after application of labeled 3MeOBA9THPP. CK standards eluted in corresponding RTs are listed together with level of detected radioactivity. (DOC) [file pone.0039293.s006.doc]

**Table S1. The retention time (RT) of radioactivity detected after application of labeled 3MeOBA9THPP.** CK standards eluted in corresponding RTs are listed together with level of detected radioactivity.

| **RT (min)** | **Standard** | root | shoot | root | shoot |
| --- | --- | --- | --- | --- | --- |
|  |  | 3-days application | | 7-days application | |
| 2-3 | Adenine | 2.66% | 4.81% | 1.93% | 4.78% |
| 4-6 | Adenosine, (OG)3OHBAP | 7.56% | 15.26% | 5.05% | 15.21% |
| 8-9 | 3MeOBAPR5MP, 3OHBAP, (OG)3OHBAP | 1.80% | 3.19% | 0.68% | 1.47% |
| 11-12 | 3MeOBAP9G, 3OHBAPR | 4.90% | 2.71% | 5.39% | 0.90% |
| 18-19 | 3MeOBAP | 2.44% | 8.57% | 2.22% | 12.42% |
| 20-21 | 3MeOBAPR | 1.75% | 0.73% | 0.43% | 0.56% |
| 23-26 | Unknown | 8.34% | 40.18% | 8.02% | 27.46% |
| 28-30 | 3MeOBA9THPP | 70.56% | 24.54% | 76.28% | 37.20% |
