# Supplementary material for: Novel Cytokinin Derivatives Do Not Show Negative Effects on Root Growth and Proliferation in Submicromolar Range
Source: PLoS One. 2012 Jun 18;7(6):e39293. doi: 10.1371/journal.pone.0039293 (PMC3377648; doi:10.1371/journal.pone.0039293)
Supplement: Table S2 — Sequences of primers used in qPCR. Accession numbers of putative proteins from Maizesequence database (http://www.maizesequence.org/index.html). Number of exons and chromosome location are listed together with GeneBank accession numbers and reference if gene has been previously characterized. Transcript abundance is expressed as gene copy number in 1ng of total RNA amplified in qPCR. (DOC) [file pone.0039293.s007.doc]

**Table S2. Sequences of primers used in qPCR.** Accession numbers of putative proteins from Maizesequence database (<http://www.maizesequence.org/index.html>). Number of exons and chromosome location are listed together with GeneBank accession numbers and reference if gene has been previously characterized. Transcript abundance is expressed as gene copy number in 1 ng of total RNA amplified in qPCR.

| Gene | Sequence of forward and reverse primers | # of exons | Protein ID | Chromosome | Reference | NCBI accession | Transcript abundance  roots leaves | |
| --- | --- | --- | --- | --- | --- | --- | --- | --- |
|  |  |  |  |  |  |  |  |  |
| LOG1 | 5´-CGCCGCATCGTCATCTC-3´ | 7 | GRMZM2G059392_T02 | 3 | This work |  | 67 ± 6 | 2382 ± 553 |
|  | 5´-TACTCCGGAACGTACTCCTCAAG-3´ |  |  |  |  |  |  |  |
|  |  |  |  |  |  |  |  |  |
| LOG3 | 5´-GCAGTCCGACGCATTCATC-3´ | 6 | GRMZM2G100360_T01 | 5 | This work |  | 117 ± 40 | 278 ± 17 |
|  | 5´-GCTGCGCCCACGTGAT-3´ |  |  |  |  |  |  |  |
|  |  |  |  |  |  |  |  |  |
| LOG4 | 5´-CTCGCCACATCATCGTCTTG-3´ | 7 | GRMZM2G122634_T01 | ? | This work |  | 17 ± 5 | < 1 |
|  | 5´-GGAGTACTCAGTCACCTCTAGCTTGTG-3´ |  |  |  |  |  |  |  |
|  |  |  |  |  |  |  |  |  |
| LOG5 | 5´-CGACCCGCTTCTGTCTTTCA-3´ | 7 | GRMZM2G073634_T01 | 6 | This work |  | 257 ± 75 | 50 ± 3 |
|  | 5´-CGCCTCCGATACGAAACCT-3´ |  |  |  |  |  |  |  |
|  |  |  |  |  |  |  |  |  |
| LOG7 | 5´-ACATGCCGACGCTTTCATC-3´ | 6 | GRMZM2G159149_T01 | 1 | This work |  | < 1 | 201 ± 13 |
|  | 5´-TCTCCAGCAGTTCTTCGATTGTC-3´ |  |  |  |  |  |  |  |
|  |  |  |  |  |  |  |  |  |
| ACS1 | 5´-CTGCTGCTAAGATTGCTCGATTT-3´ | 4 | GRMZM2G018006_T01 | 9 | This work |  | 492 ± 62 | 877 ± 192 |
|  | 5´-TGGAAACAAGCAAACGTTGTG-3´ |  |  |  |  |  |  |  |
|  |  |  |  |  |  |  |  |  |
| ACS2/7 | 5´-GTGGCGGGTCATCGTACAC-3´ | 4 | GRMZM2G040734_T01 | 10 | Gallie and Young, 2004 | AY359570 | 96 ± 11 | 54 ± 11 |
|  | 5´-GGGCTCGTTGCAGTGGAA-3´ | 4 | GRMZM2G164405_T01 | 2 | Gallie and Young, 2004 | AY359569 |  |  |
|  |  |  |  |  |  |  |  |  |
| ACS6 | 5´-GGCTCTTCTGCTGGGTCAAC-3´ | 3 | GRMZM2G054361_T01 | 1 | Gallie and Young, 2004 | AY359571 | 29 ± 6 | < 1 |
|  | 5´-CATCTCGCCCTCGAACGA-3´ |  |  |  |  |  |  |  |
|  |  |  |  |  |  |  |  |  |
| ACS15 | 5´-GGAGACGCGCTGCTGATC-3´ | 3 | GRMZM2G163015_T01 | 3 | This work |  | 149 ± 25 | < 1 |
|  | 5´-TCCTCCACCGCAGGTCTCT-3´ |  |  |  |  |  |  |  |
|  |  |  |  |  |  |  |  |  |
| ACO15/31 | 5´-AGGCCATGGACGAGAACCT-3´ | 1 | GRMZM2G166616_T01 | 10 | Gallie and Young, 2004 | AY359573 | 44 ± 7 | 388 ± 96 |
|  | 5´-CGCAGGCCCGTGATGA-3´ | 1 | GRMZM2G072529_T01 | 10 | Gallie and Young, 2004 | AY359572 |  |  |
|  |  |  |  |  |  |  |  |  |
| ACO20/35 | 5´-ACCTCCCGGCCTCCAA-3´ | 3 | GRMZM2G126732_T02 | 4 | Gallie and Young, 2004 | AY359575 | 174 ± 49 | 351 ± 62 |
|  | 5´-GGCTCCAGGCCCAGGTT-3´ | 3 | GRMZM2G052422_T01 | 5 | Gallie and Young, 2004 | AY359576 |  |  |
|  |  |  |  |  |  |  |  |  |
| ACO48 | 5´-CGTCGGCCCCACCAA-3´ | 3 | GRMZM2G013448_T04 | 8 | This work |  | 82 ± 9 | 15 ± 3 |
|  | 5´-GGTGCCAGGGTTGTAGAACGT-3´ |  |  |  |  |  |  |  |
|  |  |  |  |  |  |  |  |  |
| ACO49 | 5´-GCTGTACGTGCGGCACAA-3´ | 2 | GRMZM2G007249_T02 | 7 | This work |  | < 1 | 1260 ± 250 |
|  | 5´-TGTCCGTCTCCATGGACTTG-3´ |  |  |  |  |  |  |  |
|  |  |  |  |  |  |  |  |  |
| ACO70 | 5´-GAAGATGCTGGGCGTGATG-3´ | 3 | GRMZM2G089856_T01 | 4 | This work |  | 1014 ± 166 | < 1 |
|  | 5´-CTGACCTTGGTGCCGTAGAAG-3´ | 3 | GRMZM2G100770_T01 | 2 | This work |  |  |  |
|  |  |  |  |  |  |  |  |  |
| ACO83 | 5´-CCTATCCATCGCAACATTCTATAACC-3´ | 2 | GRMZM2G390385_T01 | 5 | This work |  | 63 ± 9 | < 1 |
|  | 5´-GCTTTGAAGCTGGGCAGATT-3´ | 3 | GRMZM2G164883_T01 | 6 | This work |  |  |  |
