# Supplementary material for: Novel Cytokinin Derivatives Do Not Show Negative Effects on Root Growth and Proliferation in Submicromolar Range
Source: PLoS One. 2012 Jun 18;7(6):e39293. doi: 10.1371/journal.pone.0039293 (PMC3377648; doi:10.1371/journal.pone.0039293)
Supplement: Table S3 — Stability of N9 -derivatives in acidic solutions. The pH stability of compounds in McIlvaine buffer with pHs decreasing from 5.5 to 4 measured 16h after sample preparation. The percentage of the released free base 3MeOBAP was determined by HPLC. (DOC) [file pone.0039293.s008.doc]

**Table S3.** **Stability of *N9* derivatives in acidic solutions.** The pH stability of compounds in McIlvaine buffer with pHs decreasing from 5.5 to 4 measured 16 h after sample preparation. The percentage of the released free base 3MeOBAP was determined by HPLC.

| pH | Compound peak area (%) | | | | | |
| --- | --- | --- | --- | --- | --- | --- |
|  | 3MeOBA9THPP | | 3MeOBA9ClBut | | 3MeOBAP9G | |
|  | *N9* derivative | Free base | *N9* derivative | Free base | *N9* derivative | Free base |
| 5.5 | 98.9 | 1.1 | 100.0 | 0.0 | 100.0 | 0.0 |
| 5.0 | 94.7 | 5.3 | 100.0 | 0.0 | 100.0 | 0.0 |
| 4.5 | 86.0 | 14.0 | 100.0 | 0.0 | 100.0 | 0.0 |
| 4.0 | 54.7 | 45.3 | 100.0 | 0.0 | 100.0 | 0.0 |
